# Molecular characterization of Newcastle disease virus obtained from Mawenzi live bird market in Morogoro, Tanzania in 2020–2021

**DOI:** 10.1007/s42770-023-01159-z

**Published:** 2023-11-01

**Authors:** John B. Tsaxra, Celia Abolnik, Terra R. Kelly, Augustino A. Chengula, James R. Mushi, Peter L. M. Msoffe, Amandus P. Muhairwa, Thandeka Phiri, Rachel Jude, Nadira Chouicha, Esther L. Mollel, Huaijun Zhou, Rodrigo A. Gallardo

**Affiliations:** 1 Department of Microbiology, Parasitology, and Biotechnology, Sokoine University of Agriculture, Morogoro, Tanzania; 2 USAID Feed the Future Innovation Lab for Genomics to Improve Poultry Project, Davis, CA USA; 3 Livestock Training Agency, Mabuki Campus, Mwanza, Tanzania; 4 Department of Production Animal Studies, Faculty of Veterinary Sciences, University of Pretoria, Pretoria, South Africa; 5 EpiEcos, Flagstaff, AZ 86004 USA; 6 Department of Animal Physiology, Biochemistry, Pharmacology, and Toxicology, Sokoine University of Agriculture, Morogoro, Tanzania; 7 Department of Veterinary Medicine and Public Health, Sokoine University of Agriculture, Morogoro, Tanzania; 8 School of Veterinary Medicine, University of California, Davis, CA 95616 USA; 9 Department of Animal Science, University of California, Davis, CA 95616 USA

**Keywords:** Newcastle disease, Genotypes, Phylogeny, Local chickens, Live bird market, Tanzania

## Abstract

Newcastle disease (ND) is among the most important poultry diseases worldwide. It is the major threat to poultry production in Africa and causes major economic losses for both local and commercial chickens. To date, half of ND class II genotypes have been reported in Africa (I, IV, V, VI, VII, XI, XIII, XIV, XVII, XVIII, and XXI). The information on the circulating NDV genotypes is still scarce despite the endemic nature of ND in most countries on the African continent.A total of 659 oro-cloacal swabs were collected from local chickens in Mawenzi live bird market located in Morogoro, Tanzania, between June 2020 and May 2021. Newcastle disease virus was detected by using reverse transcription real-time polymerase chain reaction (RT-qPCR) and conventional PCR followed by sequencing of PCR products. The prevalence of NDV in the surveilled live bird markets was 23.5%. Sequencing and phylogenetic analysis revealed the presence of sub-genotype VII.2. The detected sub-genotype VII.2 has phylogenetic links to Zambian NDV strains implying a Southeast dissemination of the virus, considering that it was first detected in Mozambique. This study underscores the need of active NDV surveillance to determine the distribution of this NDV genotype in the country and monitor its spread and contribution to the emergence of new ND viruses.

**Supplementary Information:**

The online version contains supplementary material available at 10.1007/s42770-023-01159-z.

## Introduction

Newcastle disease virus (NDV) is highly contagious, infecting both domestic and wild bird species, and causes the most economically and socially important disease of domestic poultry in Africa [1]. In susceptible chicken populations, velogenic NDV causes mortality up to 100% in affected flocks [2]. In villages of low- and middle-income countries (LMIC), such as in some rural areas in Tanzania, chickens are primarily kept in extensive scavenging systems [3], where diseases like ND serve as major constraints to poultry production [4–8]. In extensive scavenging systems, poultry from different households, ages, and species comingle with each other and sometimes encounter wild birds presenting opportunities for pathogen transmission. In these settings, some of the birds may be vaccinated against ND, while others are not [9, 10]; thereby, the inconsistency of ND vaccination can increase the risk of NDV outbreaks among village flocks. In Tanzania, indigenous chickens are mainly sold through live bird markets (LBMs), because of the lack of a cold chain to distribute chilled meat [11]. Most LBMs receive chickens, guinea fowl, and ducks from different regions of the country, making this environment conducive for the emergence and spread of viruses, such as influenza A viruses and NDVs [11].

Newcastle disease virus, an Avian Orthoavulavirus type-1 (AOaV-1) and previously known as avian paramyxovirus type-1 (APMV-1) [12], is a single-stranded negative-sense RNA (-ssRNA) virus [13]. The genome encodes six proteins namely, nucleoprotein (NP), phosphoprotein (P), matrix protein (M), fusion protein (F), hemagglutinin-neuraminidase (HN), and the RNA-dependent RNA polymerase (L) [14, 15]. All NDV strains belong to a single serotype, but there is substantial genetic and antigenic variation across strains [16, 17].

The NDV fusion (F) gene is commonly targeted for the classification of NDV into genotypes [18–20] using both partial and complete sequences [21, 22]. AOaV-1 is divided into two classes: class I and class II, with class I NDVs primarily encompassing lentogenic viruses commonly found in wild birds and less frequently in poultry. Class II NDVs consist of lentogenic (low virulence), mesogenic (medium virulence), and velogenic (highly virulent) pathotypes [23]. These strains are detected in multiple wild birds and domestic poultry species worldwide. Class I viruses have only one genotype, while class II viruses have 20 distinct genotypes [24]. In addition to genotype, the nucleotide sequence of the cleavage site of the F gene determines the pathogenicity of NDV [12, 24]. The clinical signs caused by NDV infection in chickens are variable based on the pathogenicity of the strain, and range from none (asymptomatic infection) to severe as decreased egg production, depression, diarrhea, respiratory distress, and neurological signs [24].

In Africa, a range of NDV genotypes have been reported, including genotypes I, II, IV, V, VI, VII, XI, XIII, XIV, XVII, XVIII and XXI [1, 24, 25]. In Tanzania, the first isolation and pathotyping of NDV was performed by Loretu and Mkaria [26]. More recently, researchers in Tanzania have isolated and characterized both velogenic and lentogenic NDV strains of genotypes V and XX from backyard chickens [27], and genotypes V and XIII.1.1 from live bird markets [11]. In addition, da Silva et al. [20] reported genotypes V, VII.2, and XIII in chickens. While NDV is endemic and causes devastating economic losses in indigenous chickens in Tanzania, our understanding of the diversity of NDV genotypes circulating among village poultry and in live bird market settings is still limited. Tanzania’s borders, like those of many other countries in the region, allow the relatively unrestricted trade in live chickens within the sub-region and have resulted in the spread of ND across East Africa. This study aimed to identify and molecularly characterize NDV genotypes circulating among local chickens obtained from a live bird market serving as a central poultry trading hub in Tanzania in 2021 and 2022.

## Materials and methods

### Study site

**Fig. 1:**
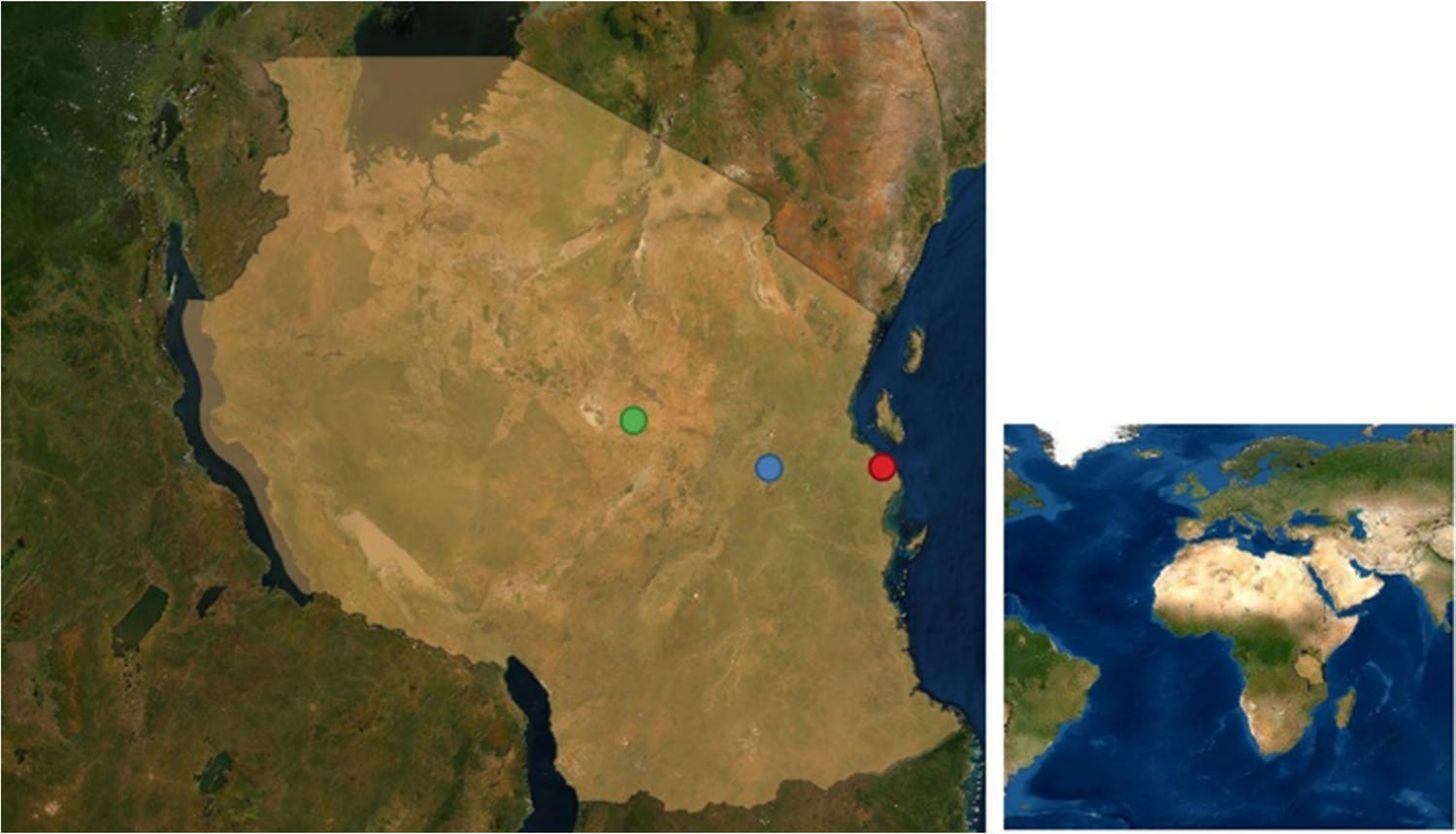
Tanzania map showing the location of Morogoro (blue dot), Dodoma (green dot), and Dar es Salaam (red dot)

The Mawenzi live bird market is in Morogoro municipality in the eastern part of Tanzania (Fig. 1). Morogoro is located 196 km west of Dar es Salaam which is the country’s largest city and commercial center, and 260 km east of Dodoma, the country’s capital city.

The Mawenzi live bird market is located within the general food market. It is an open-air market where various species of live poultry (indigenous chickens, ducks, guinea fowl) are kept in mixed-species enclosures made of wood and wire mesh and stacked on top of each other. Birds are provided with maize bran mixed with food leftovers and water. The market sells more than 300 birds (mixed species) per week. These birds originate from multiple districts within Morogoro region and other regions of the country and are transported to the market via middlemen to be sold to consumers by live bird vendors who are based at the market. The birds are collected and offloaded for sale in the market regardless of their vaccination and health status presenting challenges for NDV prevention and control. A mini slaughtering and processing area is located next to the cages, which does not have a water supply or sanitary facilities. In general, biosecurity measures are severely lacking further illustrating the potential for disease emergence and spread among birds housed in the market.

### Collection of Oro-cloacal swabs

Oro-cloacal swabs were collected from chickens at the live bird market during the period from June 2020 to May 2021. The samples were collected on a weekly basis from the first and sixth chicken from each cage. Samples were collected using sterile polyester-tipped plastic swabs (Puritan, USA). A swab was inserted in the oral cavity including the choanal cleft and back of the throat in circular motions. The same swab was then used in a circular motion against the mucosa of the cloaca. The swabs were immediately placed into a cryovial containing 0.5 mL sterile phosphate-buffered saline (PBS) and stored in a cool box before transport to at Sokoine University of Agriculture laboratory to be saved at − 80 °C.

### RNA extraction and real-time reverse transcription polymerase chain reaction (RT-qPCR)

Viral RNA was extracted from the swabs using the IndiMag Pathogen Kit in an IndiMag automated extraction instrument (Indical Bioscience, USA), following manufacturer’s instructions. Reverse transcription-quantitative polymerase chain reaction (RT-qPCR) was performed to detect the presence of the NDV, using the primers and probes described by Fuller et al. [28] that detects both class I and II AOaV-1 viruses, and VetMAX Plus RT-PCR kits (Thermo Fisher Scientific). The RT-qPCR cycling conditions consisted of a 53 °C reverse transcription for 45 min followed by one cycle at 95 °C for 15 min, and 40 cycles at 95 °C for 10 s, 50 °C for 30 s, and an extension temperature of 72 °C for 30 s. RT-qPCR was performed in a StepOne Plus thermal cycler (Applied Biosystems). Cycle threshold (Ct) values < 40 were considered positive.

### Conventional RT-PCR and Sanger DNA sequencing

In 77 samples where the Ct value after RT-qPCR was ≤ 30, a 1100 bp portion of the NDV genome that spans the 3′ end of the M gene and 5′ end of the F gene (including the F_0_ cleavage site) were amplified using primers NDV M610 (forward) and F581 (reverse) [29]. RT-PCR was also performed with a second set of oligonucleotides, Alls (forward) and Alle (reverse) that amplifies a 362 bp region of the F gene spanning the F_0_ cleavage site [30]. RT-PCR products were separated in 1% agarose gel, purified (QIAquick PCR Purification Kit, Qiagen), quantified with a Nanodrop spectrophotometer, and submitted to Inqaba Biotech (Pretoria, South Africa) for Sanger DNA sequencing.

### Ion Torrent sequencing and analysis

Transcriptomic libraries were prepared using the Sigma Whole Transcriptome Amplification Kit (Sigma, Germany), according to the manufacturer’s recommendation. DNA libraries were shipped on ice packs to the Stellenbosch University Central Analytical Facility (Stellenbosch, South Africa) for Ion Torrent sequencing. Ion Torrent reads were assembled in the CLC genomics workbench software v.22. Multiple sequence alignments of complete or partial consensus genomes were performed in MAFFT v.7. Reference partial and full NDV F gene sequences were used for classification [24] and phylogeny, including relevant sequences on the analysis. RAxML phylogenetic trees were constructed in Geneious Prime 2023.1.2 (Biomatters Ltd) using the GTR GAMMA I nucleotide model with the rapid bootstrapping and search for best scoring maximum likely hood tree algorithm using 1000 bootstrap replicates, a parsimony random seed of 456, and starting with a complete random tree [31]. The obtained sequences were uploaded to NCBI sequence read archive and can be accessed at the BioProject accession number PRJNA987660.

## Results

Six hundred and fifty-nine chicken samples were collected from the live bird market. A total of 155 (23.5%) chickens tested positive for the presence of NDV-specific RNA by RT-qPCR. Of the positive samples, 77 had cycle threshold (Ct) values less than 30 and were suitable for further genetic characterization. Using the Alls/Alle primers [30], we obtained 42 amplicons that matched the 362 bp expected fragment size. DNA was extracted from the gel and purified with the QIAquick Gel Extraction kit (Qiagen, USA), and 37 PCR amplicons were of sufficient DNA concentration for Sanger sequencing. Sequences were obtained from 27 samples; however, 18 were non-specific bacterial DNA. The nine remaining sequences were all characterized as NDV genotype VII.2 with a velogenic F_0_ cleavage site sequence of RRRKRF. Whole transcriptome libraries were prepared from these nine samples and submitted for Ion Torrent sequencing. Sequencing results are summarized in Table 1.

**Table 1:** NDV direct deep sequencing on swab samples focusing on the full NDV genome and the F gene

Sample	Complete genome^1^ (no. reads mapped) (% coverage)	F gene^2^ (no. reads mapped) (% coverage)	BLAST result*
J-122 (August, 2020)	**9806 (141) [64.5%]**	**563 (4) [33.9%]**	**Genotype VII.2**
J-145 (September, 2020)	**11,299 (189) [74.4%]**	**868 (7) [52.2%]**	**Genotype VII.2**
J-156 (September, 2020)	6101 (64) [40.2%]	153 (3) [9.2%]	Genotype VII.2
J-168 (September, 2020)	**13,220 (302) [87%]**	**1449 (22) [87.2%]**	**Genotype VII.2**
J-196 (September, 2020)	0	0	
J-219 (October, 2020)	8166 (85) [53.8%]	334 (3) [17.5%]	Genotype VII.2
J-403 (January, 2021)	1116 (12) [7.3%]	0	Genotype VII.2
J-489 (March, 2021)	**14,349 (1270) [94.5%]**	**1619 (71) [97.4%]**	**Genotype VII.2**
J-578 (April, 2021)	6,884 (64) [45.3%]	116 (1)	Genotype VII.2

^1^15192 bp reference sequence; ^2^1662 bp reference sequence; bolded samples were included in the phylogenetic analysis. *Avian Orthoavulavirus 1, isolate: Turkey/South Africa/N2057/2013 GenBank accession #: KR815908

Partial NDV genomes recovered from the swab samples varied up to 94.5% coverage. One of the issues encountered was the fragmentation of the recovered reads affecting sequencing depth and coverage. Recovery of over 30% of the F gene sequence was accomplished in four out of the nine samples. These sequences were included in the phylogenetic tree with a 598 bp segment of the F gene for comparison with previous NDVs detected in Tanzania, new relevant NDV sequences, and other genotype VII.2 references (Supplementary figure 1). The viruses from 2020 to 2021 cluster together, sharing a recent common ancestor with previously reported Tanzanian and Mozambique strains, as well as strains from Zambia and Zimbabwe from 2015 and 2013, respectively, although the bootstrap values are low.

**Fig. 2:**
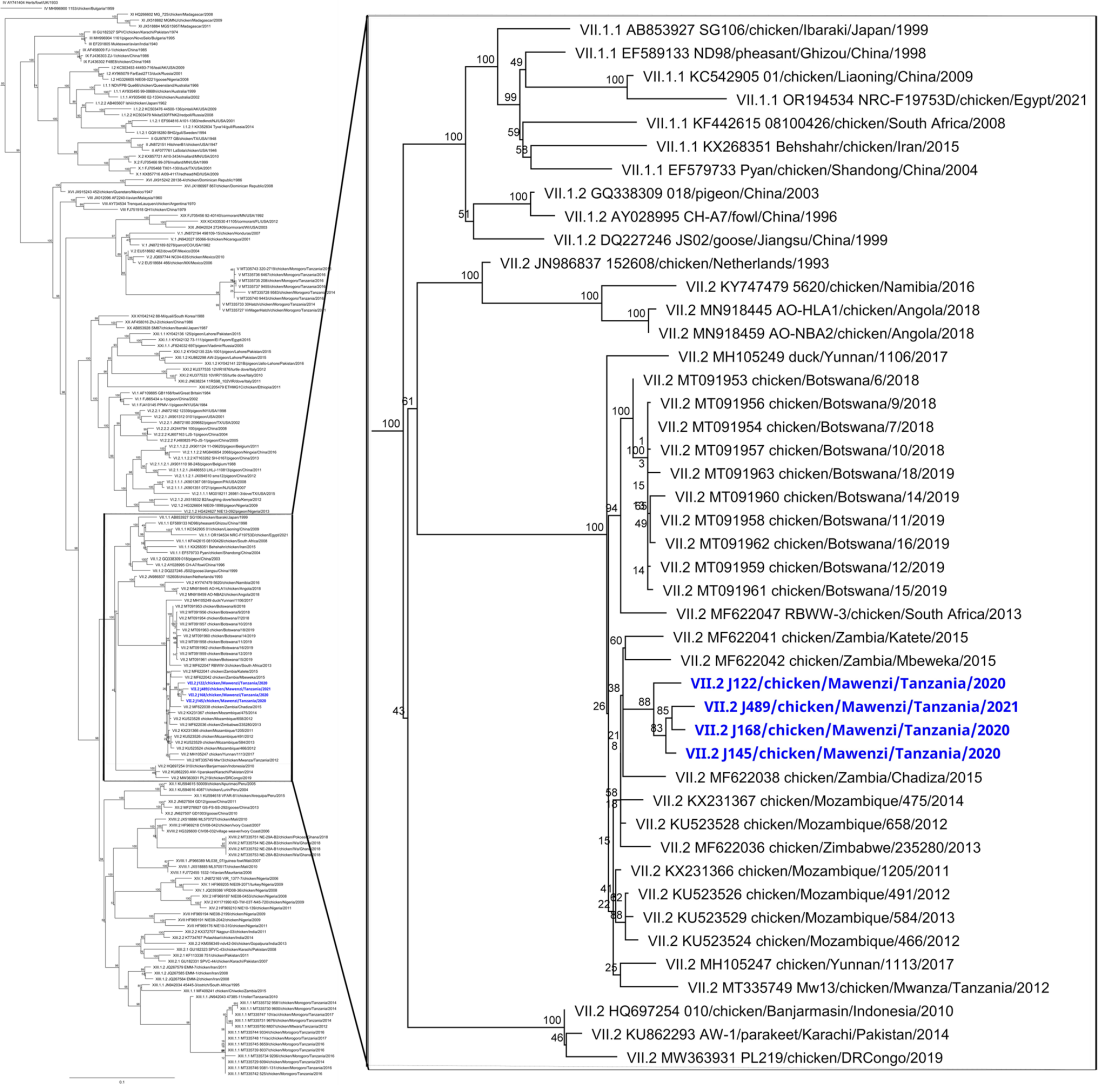
Maximum likelihood phylogenetic tree of the fusion (F) gene sequences (1687 bp). The tree is focusing on genotype VII strains. Current Tanzanian strains are highlighted in blue

To confirm the results of the phylogenetic tree reconstructed with a partial F gene sequence, we prepared a second tree using a 1687 bp segment of the F gene. Figure 2 confirms the phylogenetic grouping with strains from Tanzania, Zambia, Zimbabwe, and Mozambique. The genotype VII strain recovered from Mwanza maintains its relationship with our strains considering a larger gene sequence.

## Discussion

This study detected NDV sub-genotype VII.2 and confirmed the continued circulation of the NDV sub-genotype VII.2 in Tanzanian chickens in 2020–2021. While our sequences showed fragmentation and poor depth due to the direct sequences obtained from swabs rather than isolates, they allowed us to clearly genotype the NDV-infecting chickens in the Mawenzi market in Morogoro.

The molecular epidemiology of NDV genotype VII.2 in Africa was first investigated in 2017 [32]. At that time, the phylogenetic link of these strains with isolates obtained from Zambia was detected. The source of sub-genotype VII.2 to the African continent is thought to be the movement of infected poultry, poultry products, or fomites from Southeast Asia through Mozambique [32–34]. The other possible ways in which sub-genotype VII.2 has spread from its source in Indonesia and Malaysia to other Asian countries and to the African continent could be through the movement of wild birds [35]. Since its detection in Africa, sub-genotype VII.2 has been reported in Namibia [36], South Africa, Zimbabwe, Mozambique, Malawi, Zambia, Botswana [34], Tanzania [20], The Democratic Republic of Congo [37], and Angola [38]. Our previous report of a VII.2 strain [20] is phylogenetically linked to strains from Mozambique, Zambia, Zimbabwe, and groups in the same genotype as our current detections. Moreover, Kibasa (2020) reported the isolation of NDV genotype VII from chickens in Iringa, Tanzania, which is located at the Southern highland. The NDV obtained was 98% homologous to the virus obtained in Mozambique further confirming the porosity of the borders. These countries are near Tanzania suggesting the virus spread due to proximity. Sequences from Botswana and Yunnan (China) are recent additions to GenBank and help explain the potential distribution of these viruses not only in Africa but also in Asia. A meta-analysis reported by Mngumi et al. [1] categorizes sub-genotype VII.2 as a widespread NDV genotype in Africa with reports in East, West, South, and Central African countries. In Tanzania, vaccination of chickens against ND is the key to fighting the Newcastle disease as in many other countries worldwide. The vaccination practices in local indigenous chickens are low and irregular as compared to the commercial chickens which partly results from limited access to veterinary services contributing to the emergence and maintenance of viruses in the poultry populations [39]. In addition, the mix of poultry species and population and movement through the live bird markets perpetuate and maintain the virus [40]. Although chickens are vaccinated, sub-genotype VII.2 has been implicated to have the ability to cause outbreaks [41]. This may happen due to inadequate vaccinations which results in inadequate immune responses or concurrent infection with immunosuppressive agents which compromise the mounting of adequate immune response [2].

NDV genotype VII has been of global economic importance due to its diverse nature and recurrent outbreaks in Eastern Europe, the Middle East, and Asia and sporadic outbreaks in Africa and South America [21]. This genotype is the virus responsible for the fifth NDV panzootic [37, 42–44]. The panzootic nature of sub-genotype VII.2 was predicted [21, 45] due to its nature and rapid spread from its source in Indonesia, to Pakistan, Israel, and Eastern Europe [46]. The isolation of sub-genotype VII.2 in Tanzania suggests the spread of this virus from neighboring countries and is evidence of the porosity of the country’s borders. Biosecurity measures at this level including but not limited to poultry and poultry product import regulations might help reduce the permeability of the borders and protect the country’s poultry health status. In addition, the live bird market dynamics including the long distances traveled by birds to be sold at live bird markets and the lack of biosecurity along this commute contributes to virus dissemination. Biosecurity improvements and continued surveillance would help limit dissemination and improve our understanding of the geographical distribution of this genotype and others. In addition, it will inform the establishment of control measures to limit the spread and subsequently reduce losses caused by NDV. This study contributes to the understanding of the circulating NDV strains in Tanzania.

### Supplementary Information

Below is the link to the electronic supplementary material.

Supplementary file1 (PNG 2637 KB)

## Data Availability

All the data generated during this study are included in this manuscript.
