# Supplementary figures and images for: Molecular characterization of Newcastle disease virus obtained from Mawenzi live bird market in Morogoro, Tanzania in 2020–2021
Source: Braz J Microbiol. 2023 Nov 1;54(4):3265–73. doi: 10.1007/s42770-023-01159-z (PMC10689586; doi:10.1007/s42770-023-01159-z)

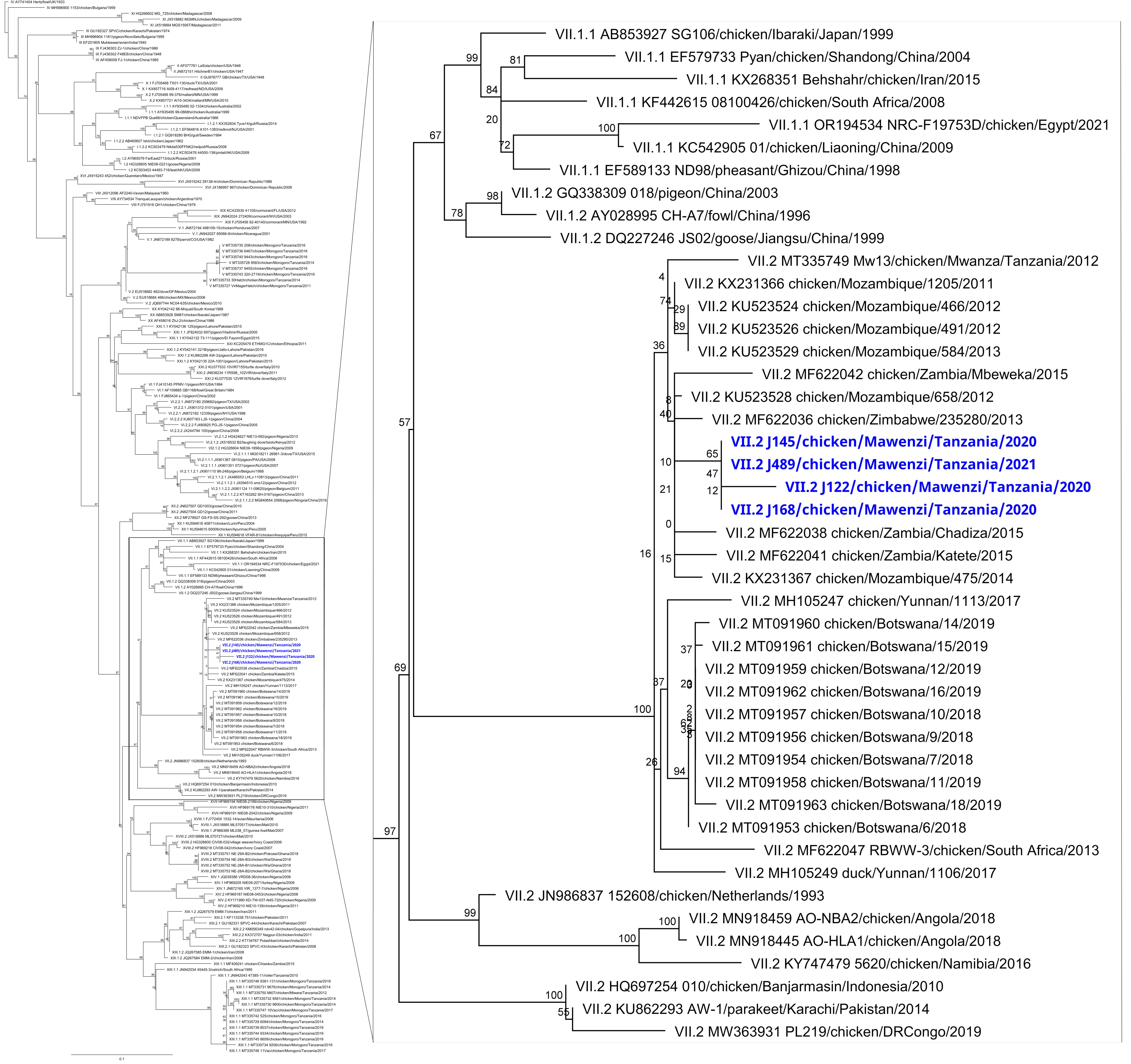

Supplement: Supplementary file 1 — Supplementary file1 (PNG 2637 KB) [file 42770_2023_1159_MOESM1_ESM.png]
